# Certification as dysplasia unit and its impact on large loop electrosurgical excision (LEEP)

**DOI:** 10.1007/s00404-022-06807-7

**Published:** 2022-10-08

**Authors:** Tatjana Hanczuk, Martin Weiss, Leon Henes, Tobias Engler, Felix Neis, Melanie Henes

**Affiliations:** 1 Department of Women’s Health, University Hospital Tübingen, Calwerstraße 7, 72076 Tübingen, Germany; 2 Eberhard Karls University of Tübingen, Tübingen, Germany

**Keywords:** Cervical intraepithelial neoplasia, Dysplasia, Colposcopy, Quality assessment

## Abstract

**Purpose:**

This study evaluates the overall treatment indicators and outcomes of patients who underwent loop electrosurgical excision procedure (LEEP) at the Department of Women’s Health Tübingen and the impact of certification as a dysplasia unit on treatment quality.

**Methods:**

Retrospective data analysis of 1596 patients from 2013 to 2018 who underwent LEEP excision at the Department of Women’s Health Tübingen. Data of cytology, colposcopy, biopsy, LEEP histology, repeat LEEP histology and general characteristics were collected and analyzed descriptively.

**Results:**

85.4% (1364) of patients had CIN 2 + and 14.6% (232) had CIN 1 or normal findings on LEEP histology. The proportion of CIN 2 + excisions increased significantly from 82.4% in 2013 to 89% in 2018. The concordance of HSIL biopsy and LEEP histology was 89.1% in 2013 and 92.6% in 2018. In 2018, more biopsies and colposcopies were performed before excision. Complete resection (R0) was achieved in 88.3% of all excisions. R0 rates in patients with CIN 3 increased in 2014–2017 compared to 2013, resulting in fewer Re-LEEP excisions and hysterectomies.

**Conclusion:**

Certification as a dysplasia unit and the associated requirements have improved the diagnostic quality for patients with cervical dysplasia undergoing LEEP. This was demonstrated by several treatment indicators such as the number of colposcopies and biopsies and treatment outcomes such as an increased proportion of CIN 2 + excisions and R0 resections.

## What does this study add to the clinical work

**Table:** 

The certification as dysplasia unit in 2014 has significantly improved treatment indicators and outcome measures for patients who underwent loop electrosurgical excision procedure (LEEP) at the Department of Women’s Health Tübingen. By complying with quality standards, the certification guarantees high quality and safety for patients undergoing LEEP.	

## Introduction

Cervical cancer is still one of the most common cancers, with approximately 600,000 new cases worldwide in 2020, and is responsible for 3.3% of cancer death rate [1]. In Germany, cervical cancer morbidity and mortality rates have decreased significantly over the past 30 years, not least due to cytological screening, consisting of an annual PAP smear, introduced in 1971 [2]. The incidence of cervical cancer is expected to decline in the future due to increasing HPV vaccination [3, 4]. If high-grade cervical intraepithelial neoplasia (CIN) is suspected, loop electrosurgical excision procedure (LEEP) may be performed to treat the CIN and prevent its development into cervical cancer. Colposcopy and, if necessary, biopsy should always be performed before surgery [2]. The patient’s human papillomavirus (HPV) status and risk factors such as age, smoking, hormonal contraception, or immunosuppression should also be considered before performing LEEP to avoid over treatment [5–7]. To maintain and verify the high-quality standards, the Department of Women’s Health Tübingen was certified as a dysplasia unit in 2014. This certification resulted from collaboration of the Cervical Pathology and Colposcopy Committee (AG CPC), the German Society of Gynecology and Obstetrics (DGGG), the German Cancer Society (DKG) and the Working Group on Gynecological Oncology (AGO) and has certain requirements, for example scientific research, performance of at least 300 colposcopies, of which 100 per examiner, and 100 LEEP per year, documentation of the procedures and outcomes, and an audit with on-site inspection every three years. Documented excisions should meet certain criteria, for example, more than 85% of LEEP specimens should have at least a CIN 2, 80% complete removal (R0) of CIN 3, or performance of a colposcopic examination before each LEEP. Acquisition of a colposcopy diploma and regular training is required for medical specialists working in a dysplasia unit as appointed examiners [8, 9].

The aim of this study was to evaluate treatment indicators and outcomes using data from patients who underwent LEEP at the Department of Women’s Health Tübingen between 2013 and 2018 and to assess whether certification has improved the quality.

## Methods

### Study design

This is a retrospective, monocentric data analysis. Ethics approval was obtained by the ethics review board of the University Hospital Tübingen. Following the ethics approval, obtaining informed consent from the patients was not required. All data were analyzed anonymously. The data were taken retrospectively from the medical record; there was no patient re-contact for the study.

The data analysis was based on all patients who underwent *colposcopy-guided* LEEP in the dysplasia unit at the Department of Women’s Health Tübingen between 2013 and 2018. Records of excisions due to the histological result of a first LEEP in the same patient were excluded. Patient characteristics such as HPV status, age, BMI, menopausal status, contraception, number of children, and smoking status were recorded. Colposcopic impression (IFCPC 2011 classification) with transformation zone (TZ) types 1–3, *colposcopy-guided* Pap smear results (Munich II classification before 2015, Munich III after 2015), number and histology of biopsy, and histologic results of LEEP with complete (R0) or incomplete (R1) resection and possibly repeated LEEP or hysterectomy were also recorded. Especially in young patients with incomplete family planning, tissue excision was performed with restraint to preserve as much cervical tissue as possible. Resection status was determined histologically. According to the guideline, the follow-up was performed by a gynecologist in private practice and the patients were only seen again if there were any abnormalities. Patients with higher grade dysplasia and incomplete resection were referred in-house for close colposcopic follow-up. Histology of biopsy and LEEP was assessed in patients with no dysplasia, CIN 1–3, ACIS, and cervical carcinoma. For the trend from 2013 to 2018, the years 2013 and 2018 were also compared directly with each other.

### Grouping of histology

For interrater reliability (Cohen's kappa) and comparison of biopsy and LEEP histology, we examined the following groups: no dysplasia, CIN 2 + (CIN 2, CIN 3, and squamous cell carcinoma), and ACIS + (ACIS and adenocarcinoma).

For the other descriptive analyses, the histology was divided into the groups: no dysplasia/CIN 1 and CIN 2 + (CIN 2, CIN 3, ACIS and Carcinoma). Carcinoma was only referred to a histological group in the LEEP histology because no carcinoma was present in the biopsies. Underdiagnosis was present when the biopsy showed no dysplasia/CIN 1 and LEEP histology was CIN 2 + . Overdiagnosis was present when the biopsy showed CIN 2 + and LEEP histology was no dysplasia/CIN 1. Concordance was present when the histological groups of biopsy and LEEP excision were the same.

### Statistics

Statistical methods were descriptive, chi-square test, Cohen's kappa and were performed in IBM SPSS Statistics 25 with a significance level of *p* < 0.05.

## Results

### Overall

Between 2013 and 2018, a total of 1596 patients underwent LEEP at the Department of Women’s Health Tübingen. The mean age was 36.6 years (± 9.83) and 8.4% (*n* = 134) were postmenopausal at the time of LEEP. A total of 85.4% (n = 1364) had CIN 2 + and 14.6% (*n* = 232) CIN 1 or normal findings in the LEEP histology (Table 1). Table 1 also shows the respective cytology in-house to the LEEP histology. The higher the grade of the lesion, the more likely a group IVa Pap was present (37.9% for CIN 2, 66.7% for CIN 3, and 83.7% for cancer). This compares to 48.1% for ACIS.

**Table 1:** LEEP histology (*n* = 1596) and comparison with the in-house cytology if a colposcopy-guided Pap smear was performed before LEEP (*n* = 1565)

	Total *n|%*	LEEP histology *n|%*					
		No dysplasia	CIN 1	CIN 2	CIN 3	ACIS	Cancer
		129|8.1	103|6.5	259|16.2	1007|63.1	53|3.3	45|2.8
Cytology in-house	I, IIa	19|15.6	10|9.9	10|3.9	31|3.1	0	0
	II	7|5.7	0	5|2.0	12|1.2	2|3.8	1|2.3
	IIg, IIp	6|4.9	6|5.9	6|2.3	9|0.9	2|3.8	0
	III, IIIg	7|5.7	1|1.0	3|1.2	18|1.8	8|15.4	2|4.7
	IIIp	12|9.8	14|13.9	20|7.8	57|5.8	7|13.5	0
	IIID	17|13.9	9|8.9	21|8.2	52|5.2	0	0
	IIID1	10|8.2	18|17.8	26|10.2	37|3.7	1|1.9	0
	IIID2	13|10.7	28|27.7	68|26.6	109|11.0	4|7.7	0
	IVa, IVag, IVap	29|23.8	15|14.9	97|37.9	661|66.7	25|48.1	36|83.7
	IVb, IVbp, IVbg	1|0.8	0	0	3|0.3	2|3.8	2|4.7
	V, Vp	1|0.8	0	0	2|0.2	1|1.9	2|4.7

**Fig. 1:**
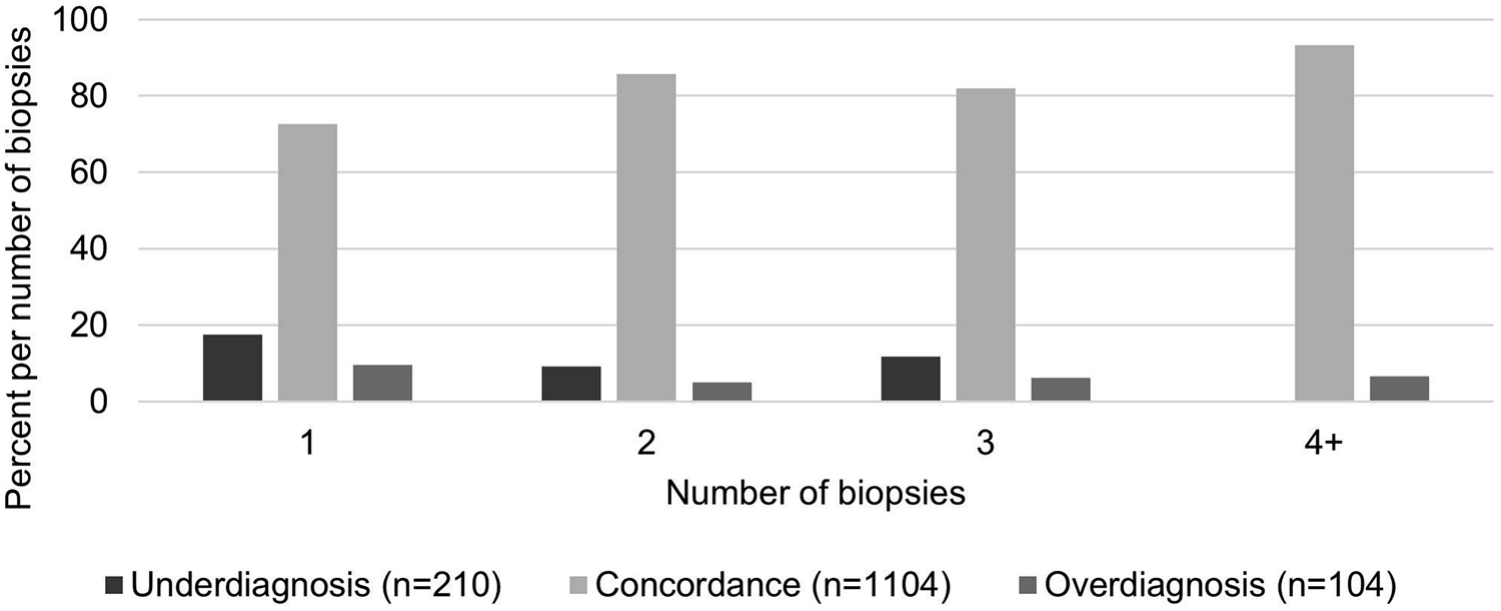
Number of biopsies taken ande rate of agreement between biopsy and LEEP histology (*n* = 1418). Histology results were classified: no dysplasia/CIN 1 and CIN 2 + (CIN 2, CIN 3, ACIS and Carcinoma). Underdiagnosis was present when the biopsy showed no dysplasia/CIN 1 and LEEP histology resulted in CIN 2 + (*n* = 210). Overdiagnosis was present when the biopsy showed CIN 2 + and LEEP histology resulted in no dysplasia/CIN 1 (*n* = 104). Concordance was present when the histological results of a biopsy and LEEP excision were the same (*n* = 1104)

Interrater agreement kappa between biopsy and cone histology showed a fair agreement with *κ* = 0.35 (*p* < 0.001). As shown in Table 2, the agreement between biopsy and cone histology increased with the severity of CIN (30.1% for CIN 1, 90.3% for CIN 2 +) and with the number of biopsies taken (72.7% for one biopsy, 85.7% for two biopsies, 93.3% for four or more biopsies) (Fig. 1).

**Table 2:**
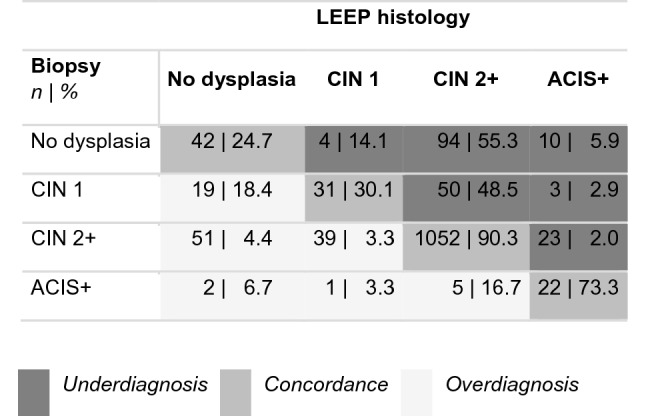
Comparison of biopsy histology and the following LEEP histology (*n* = 1468). Compared were the groups no dysplasia, CIN 1, CIN 2 + (CIN 2, CIN 3, and squamous cell carcinoma) and ACIS + (ACIS and adenocarcinoma)

When considering patients with CIN 2 + biopsies and comparing them with LEEP histology, divided into two groups (normal/CIN 1 and CIN 2 +), agreement with LEEP histology increased with visibility of the TZ (92.6% at T1, 86.2% at T2, 83.2% at T3) (Table 3) and with an abnormal colposcopic impression (90.3% vs. 82.1% normal colposcopic impression). In 94.3% of patients with T3, biopsy was performed and 67.9% also received endocervical curettage (ECC). In 16 patients (5.7%) with T3, LEEP was performed directly without biopsy/ECC, 11 of them had Pap IV or V and 5 patients had recurrent IIID or, after 2015, IIID2 Pap smears. R0 resection was achieved in 88.3% (*n* = 1294) of all excisions, and 6.1% (*n* = 98) received repeated LEEP. In patients with CIN 2 or CIN 3, R0 resection was performed in 91.1% (*n* = 1134) of the cases, compared with 71.7% (*n* = 38) for ACIS. 46% (150) of patients with R1 resection status underwent direct re-LEEP or hysterectomy, 30.8% (48) of patients failed to attend recommended check-ups, and 23 patients (21.3%) canceled check-ups prematurely.

**Table 3:** Concordance, over- or underdiagnosis rate of patients with a high-grade biopsy (CIN 2, CIN 3, and ACIS) and LEEP histology based on the transformation zone (TZ) type (*n* = 1178). Concordance was present when the LEEP histology was CIN 2, CIN 3 or ACIS (*n* = 1060). Overdiagnosis was present when the LEEP histology showed no dysplasia or CIN 1 (*n* = 89). Underdiagnosis was present when the LEEP histology showed a cervical carcinoma (*n* = 29)

	High-grade biopsy and LEEP histology		
TZ *n|%*	Overdiagnosis	Concordance	Underdiagnosis
Type 1	38|4.9	712|92.6	19|2.5
Type 2	30|11.9	218|86.2	5|2.0
Type 3	21|13.5	130|83.2	5|3.2

### Trend

On average, there were 266 excisions per year, the fewest in 2014 (*n* = 212), and the most in 2017 (*n* = 308).

**Fig. 2:**
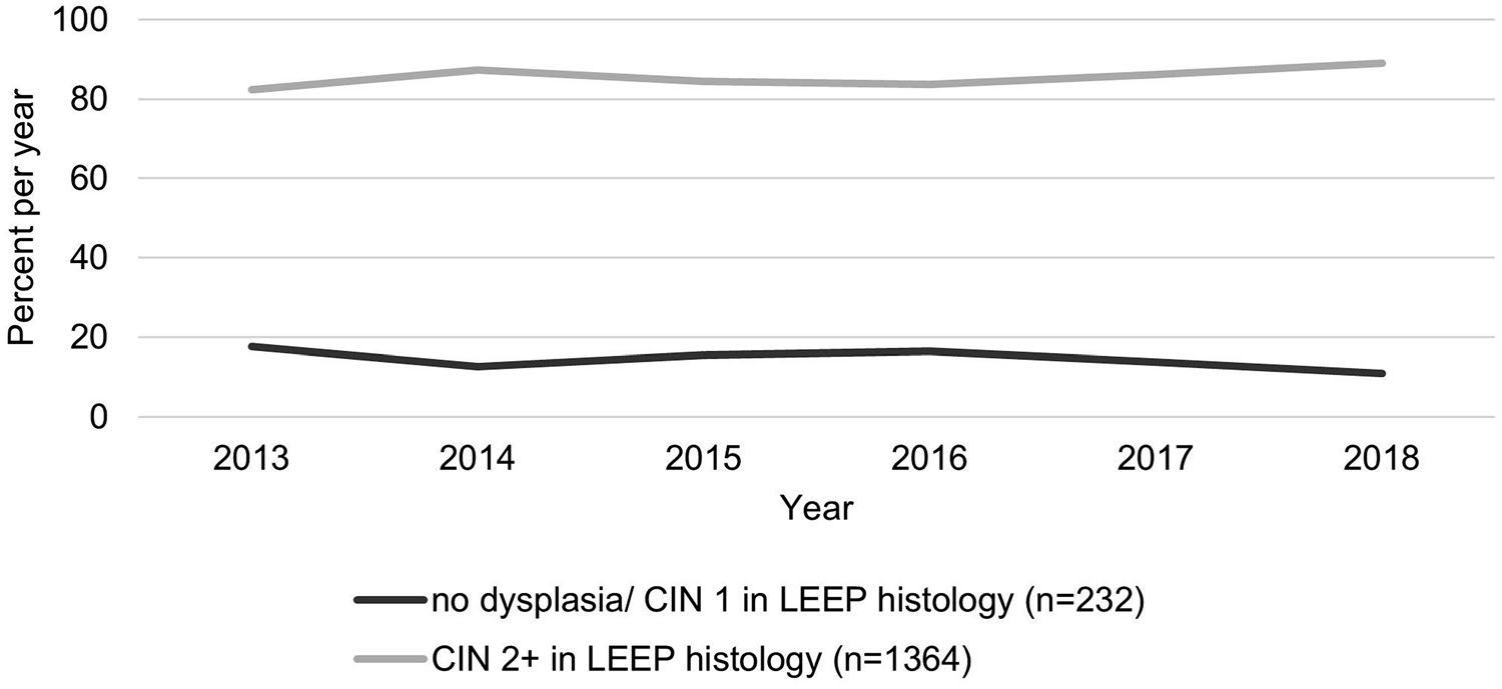
Rate of LEEP histology per year for the groups: no dysplasia/CIN 1 (*n* = 232) and CIN 2 + (CIN 2, CIN 3, ACIS and Carcinoma) (*n* = 1364)

Over the years, the proportion of CIN 2 + excisions increased significantly (*p* < 0.001) from 82.4% (*n* = 211) in 2013 to 89% (*n* = 251) in 2018 (Fig. 2).

**Fig. 3:**
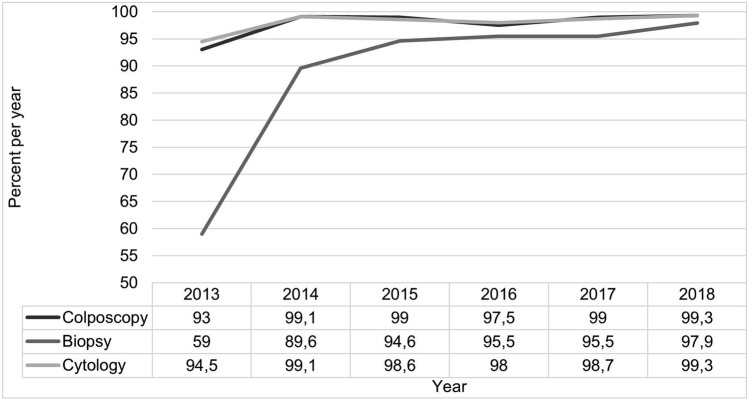
Rate of colposcopies (*n* = 1562), biopsies (*n* = 1422) and cytology (*n* = 1565) performed in-house before LEEP excision per year between 2013 and 2018

Concordance of CIN 2 + biopsy and LEEP histology was 89.1% (*n* = 98) in 2013 and 92.6% (*n* = 214) in 2018. Patients with a CIN 2 + LEEP had a CIN 2 + biopsy in 78.1% (*n* = 100) in 2013 and 87.3% (*n* = 219) in 2018 (*p* = 0.021). Significantly more biopsies (97.9% (*n* = 276) in 2018 vs. 59% (*n* = 151) in 2013, *p* < 0.001), colposcopies (99.3% (*n* = 280) in 2018 vs. 93% (*n* = 238) in 2013, *p* = 0.002), and pap smears (99.3% (*n* = 280) in 2018 vs. 94.5% (*n* = 242) in 2013, *p* < 0.001) were performed in-house in 2018 compared to 2013 prior to excision (Fig. 3).

In 2018, 100% (*n* = 282) of the patients received biopsy and colposcopy before LEEP, including the known ex domo examination results. The proportion of patients with known HPV status has increased significantly over the years, from 48.4% (*n* = 124) in 2013 to 61% (*n* = 172) in 2018 (*p* = 0.003). In 2013, the R0 proportion was 81.9% (*n* = 181), with a minimum of 89.2% (*n* = 249) and a maximum of 92.6% (*n* = 214) in the subsequent four years. R0 rates in patients with at least CIN 2 (CIN 2 +) increased in 2014–2017 (minimum 89.1% (*n* = 236) in 2017, maximum 91.6% (*n* = 186) in 2016) compared with 81% (*n* = 171) in 2013, resulting in fewer hysterectomies and, except for 2018, fewer repeat LEEP (Table 4).

**Table 4:** Rate of complete resection (R0) of the lesion per year for patients with CIN 2 + LEEP histology (CIN 2, CIN 3, and carcinoma) (*n* = 1193). Number and percentage of patients who received a repeated LEEP (*n* = 98) and patients who underwent hysterectomy (*n* = 88) per year. Number and percentage of CIN 2 + repeated LEEP (*n* = 31) and CIN 2 + Hysterectomy (n = 26)

Year *n|%*	R0 with CIN 2 + LEEP	Repeat LEEP	Hysterectomy	CIN 2 + in repeated LEEP	CIN 2 + in Hysterectomy
2013	171|81.0	19|7.4	25|9.8	7|2.7	7|2.7
2014	166|89.7	10|4.7	8|3.8	2|0.9	4|1.2
2015	221|89.5	14|4.8	12|4.1	6|2.0	4|1.4
2016	186|91.6	12|4.9	9|3.7	5|2.0	3|1.2
2017	236|89.1	19|6.2	9|2.9	5|1.6	3|1.0
2018	213|84.9	24|8.5	25|8.9	11|3.9	8|2.8

## Discussion

Defining new standards and certification of specialized units always aim on improving patients safety. The effect of such standardization needs to be supervised and examined regularly. With 1591 patients, this study represents a large cohort. Over 85% (1364) of these patients had at least CIN 2 + in the LEEP histology, which complies with the requirements of the European Federation of Colposcopy (EFC) and the AG CPC [8], and several studies that reported 68–74% CIN 2 + excisions [10–12]. The interrater agreement between the biopsy and the LEEP histology only showed a fair agreement (*κ* = 0.35), however, two different procedures (biopsy and LEEP) were compared here. A meta-analysis by Underwood et al., was able to show that the sensitivity of a CIN 2 + biopsy to detect CIN 2 + in excision is 80.1%, but the specificity is only 63%. The high sensitivity would be caused by verification bias, as patients with negative biopsies usually do not receive excision [13]. This should also be considered here, as only patients who received LEEP were included in this analysis. Other studies have described different reasons for the discrepancy between biopsy and LEEP. In our patients, the agreement between biopsy and LEEP was higher in high-grade lesions (HSIL) and in correlation to the number of biopsies taken. More biopsies are associated with a higher agreement with LEEP histology [11, 13–15]. However, Zuchna et al. described that three biopsies were not associated with higher sensitivity but would be performed primarily in women with fully visible SCJ, making the lesion obvious in these patients [15]. Another reason against performing more than two biopsies is pain resulting in anxiety and stress, which may lead to lower patient compliance [16]. Only 15 patients (1%) in our study had four or more biopsies with 86.7% concordance. The majority of patients had two biopsies (67.7%) with a concordance of 85.7%, which was significantly higher than one biopsy (67%). Of the 178 (12.3%) patients with three biopsies, 132 (74.2%) had a concordance with the LEEP histology. The visibility of the lesion is also important for its detection by colposcopy or biopsy. In patients with a type 3 TZ, the agreement between biopsy and LEEP was significantly lower than in patients with a fully visible TZ. Other studies also have shown that a type 3 TZ correlates with underdiagnosis of biopsies [12, 17]. Patients with type 3 TZ are usually older [18], and with increasing age, diagnostic accuracy in detecting a high-grade lesion decreases [19, 20]. Therefore, endocervical curettage should be performed in type 3 TZ [2]. In a recent review, endocervicoscopy was found to be a helpful diagnostic tool to assess endocervical lesions in patients with nonvisible TZ [21]. More than 88% of our patients had complete resection of the lesion, and only 9% with CIN 2 or CIN 3 had R1 resection on LEEP histology. A meta-analysis by Arbyn et al. showed that R1 rates are on average 23.1% (with a vast range of 2.8–59.5%), with almost no study being below the 20% R1 resection rate as required by the EFC [22]. Achieving R0 in all cases would often require a deeper and larger excision, which in turn may be associated with an increased risk of subsequent pregnancy complications [23, 24]. Older age is associated with a lower probability of spontaneous regression, involved endo- or endo- and ectocervical margin, and persistent high-risk HPV infection. This also increases the risk of recurrence or residual lesions [22, 25–27]. Therefore, resection status alone is not the only factor predicting treatment failure. Close follow-up examinations are recommended for these patients at a certified dysplasia unit.

Looking at the treatment indicators, there has been a positive development over the years. A colposcopic examination before LEEP in all cases is considered a quality characteristic of a dysplasia unit and is also required by the EFC [8, 28]. This requirement has continuously increased from 93% in 2013 to 99.3% in 2018. In addition, the percentage of in-house performed cytology has increased from 94.5% to 99.3%. The largest increase was achieved in biopsies performed before LEEP: while in 2013 it was only 59%, in 2018 97.9% had received a biopsy. When adding the known ex domo examination results, all patients received a biopsy and a qualified colposcopy before the excision in 2018. Also, more CIN 2 + biopsies were performed. Thus, the indication for LEEP could be made more frequently based on the synopsis of the different findings from biopsy, colposcopy, and cytology. Furthermore, the agreement rates of CIN 2 + biopsies and CIN 2 + LEEP could be increased significantly. While it was already 78.1% in 2013, 87.3% of patients with a CIN 2 + LEEP also had a CIN 2 + biopsy in 2018. A prospective study by Luyten et al., with 10,896 patients, involving eight German hospitals from 2008 to 2014, examined the implementation and application of EFC quality standards in routine colposcopy. Colposcopy before excision was performed in an average of 94.3% of patients. Direct excision was justified partly by externally performed colposcopy [29]. The annual report of certified gynecological dysplasia units presents metrics of requirements from 2014–2018 for all dysplasia units, including metrics from only seven certified units in 2014 versus 35 certified dysplasia units in 2018. The metrics for the Department of Women’s Health Tübingen are included in an annual report. All units were able to meet the colposcopy requirements each year [30].

Knowledge of the HPV high-risk (HR) status may support the indication for LEEP or a conservative approach in individual cases [31, 32]. The proportion of patients with known HPV HR status has increased significantly over the years. With the new screening introduced in Germany in 2020, consisting of a PAP smear and HPV testing every three years for women over 35 years of age, this proportion will continue to increase in the future [33]. Until 2020 there was no regular HPV testing and especially in the case of recurrent PAP IIID2 and IVa or other high-grade changes, the indication for colposcopy was often made directly.

There were also significant changes in treatment outcomes after certification as dysplasia unit. The rate of CIN 2 + excisions was already 82.4% in 2013 and steadily increased to 89% of patients with CIN 2 + in 2018. This is more than the 85% CIN 2 + excisions required by the EFC and AG CPC [9, 28]. This quality characteristic was met in the prospective study by Luyten et al. with an average of 86.4% [29]. It is reasonable that this requirement may need to be reevaluated in the future due to the new screening method including HPV testing and due to higher vaccination rates resulting in an overall lower HPV and thus a lower CIN 2 + prevalence [29].

*Compared to 2013, the proportion of R0 resections had been consistently higher in subsequent years,* but as previously described, resection status alone is not an appropriate criterion to ensure the long-term success of LEEP. Because of the higher R0 rates, hysterectomies and, except for the year 2018, re-LEEP were less frequent. In the study of Luyten et al., this criterion was criticized and only a few of the participating hospitals achieved this quality measure. Reasons for this might be the lack of documentation or that control examinations and HPV testing were preferred to the resection status [29]. In the annual report of the certified dysplasia units, the reporting of more than 80% CIN 3 resections in 2018 had a wide range between 62–100%. The median for the respective years was stable at approximately 85%, and the different units specified almost similar reasons for not reaching this target, such as tissue-sparing excision in the case of incomplete family planning [30].

## Conclusion

Treatment indicators and outcome measures for patients with LEEP at the Department of Women’s Health Tübingen have improved significantly over the years due to the certification as dysplasia unit according to AG-CPC. The indication for LEEP could be made more reliable based on several factors and their synopsis, thus avoiding overtreatment. The precise documentation of case numbers enables to identify unmet goals and continuous clinical improvement. Complying with quality standards, participating in regular training courses, and conducting further research, certification guarantees high quality and safety for patients in case of indication, implementation, and follow-up of LEEP.
